# Evaluation of the precision of navigation-assisted endoscopy according to the navigation tool setup and the type of endoscopes

**DOI:** 10.1007/s00701-022-05276-w

**Published:** 2022-06-28

**Authors:** Lara Chavaz, Alioucha Davidovic, Torstein R. Meling, Shahan Momjian, Karl Schaller, Philippe Bijlenga, Julien Haemmerli

**Affiliations:** 1 Faculty of Medicine, University of Geneva, Geneva, Switzerland; 2 Division of Neurosurgery, Department of Clinical Neurosciences, Geneva University Hospitals, Rue Gabrielle-Perret-Gentil 4, 1205 Geneva, Switzerland

**Keywords:** Navigation-assisted endoscopy, Neuronavigation, Neuroendoscopy, Neurosurgery, Endoscopy

## Abstract

**Object:**

Preoperative image-based neuronavigation-assisted endoscopy during intracranial procedures is gaining great interest. This study aimed to analyze the precision of navigation-assisted endoscopy according to the navigation setup, the type of optic and its working angulation.

**Methods:**

A custom-made box with four screws was referenced. The navigation-assisted endoscope was aligned on the screws (targets). The precision on the navigation screen was defined as the virtual distance-to-target between the tip of the endoscope and the center of the screws. Three modifiers were assessed: (1) the distance *D* between the box and the reference array (CLOSE 13 cm – MIDDLE 30 cm – FAR 53 cm), (2) the distance between the tip of the endoscope and the navigation array on the endoscope (close 5 cm – middle 10 cm – far 20 cm), (3) the working angulation of the endoscope (0°-endoscope and 30°-endoscope angled at 90° and 45° with the box).

**Results:**

The median precision was 1.3 mm (Q1: 1.1; Q3: 1.7) with the best setting CLOSE/close. The best setting in surgical condition (CLOSE/far) showed a distance-to-target of 2.3 mm (Q1: 1.9; Q3: 2.5). The distance *D* was correlated to the precision (Spearman rho = 0.82), but not the distance *d* (Spearman rho = 0.04). The type of optic and its angulation with the box were also correlated to the precision (Spearman rho =  − 0.37). The best setting was the use of a 30°-endoscope angled at 45° (1.4 mm (Q1: 1.0; Q3: 1.9)).

**Conclusion:**

Navigated-assisted endoscopy is feasible and offers a good precision. The navigation setup should be optimized, reducing the risk of inadvertent perifocal damage.

## Introduction

Endoscopy is gaining interest in neurosurgery, not only during endonasal approaches, but also for intracranial procedures [6, 10, 15, 23, 25, 26, 29]. Its main advantage over open procedures resides in its minimal invasiveness with less postoperative morbidity, especially to reach deep-seated brain lesions, skull base surgery or to open basal cisterns in case of hydrocephalus [4, 6, 23, 25]. In some selected neurosurgical indications, straight rigid and flexible endoscopes were reported to be suitable to, for example, perform ventriculocisternostomies and present the same rate of complications [2, 19]. These two techniques, however, harbor certain limitations in surgical and anatomical visualization: the surgeon has to work with a non-stereoscopic view and a lack of depth perception during endoscope-assisted procedures [20, 28], despite novel high-definition cameras [12]. Even well-trained endoscopic neurosurgeons may experience loss of anatomical landmarks during endoscopic surgery, especially in case of bleeding, abscess or in tumoral cases.

Based on preoperative CT or MR images, standard neuronavigation (NV) systems allow the surgeon to permanently and intraoperatively localize anatomical structures [7, 16]. The possibility to reference the straight rigid endoscope as a navigated tool has been described and is used in daily practice to overcome the limitations of endoscopy [1, 5, 8, 11, 15, 24, 29]. The endoscope and its tip are then visualized on dedicated 2D navigation screens, allowing the surgeon to anticipate and to reduce unwanted perifocal lesions. The technique is reported as reliable, safe and precise [1, 8, 11]. However, because of its mobility, flexible endoscopes cannot be navigated.

Navigation-assisted endoscopy requires, however, at least one reference array fixed to the skull clamp and one navigation array, which is solidary to the endoscope. To the best of our knowledge, no study has analyzed the influence of the positions of the reference and navigation arrays with respect to the precision during intracranial endoscopy. Furthermore, no study has reported the influence of the type of endoscope and its angulation of use on the precision.

This study aims to analyze the precision of navigation-assisted endoscopy according to the navigation tool setup and according to the type of endoscopes and their use. Using a custom-made model, we hypothesized (1) that the precision (distance-to-target) was better when the reference and navigation arrays were close to the target and (2) that the precision varies with the angle between the endoscope and the target.

## Methods

### Custom-made model

**Fig. 1:**
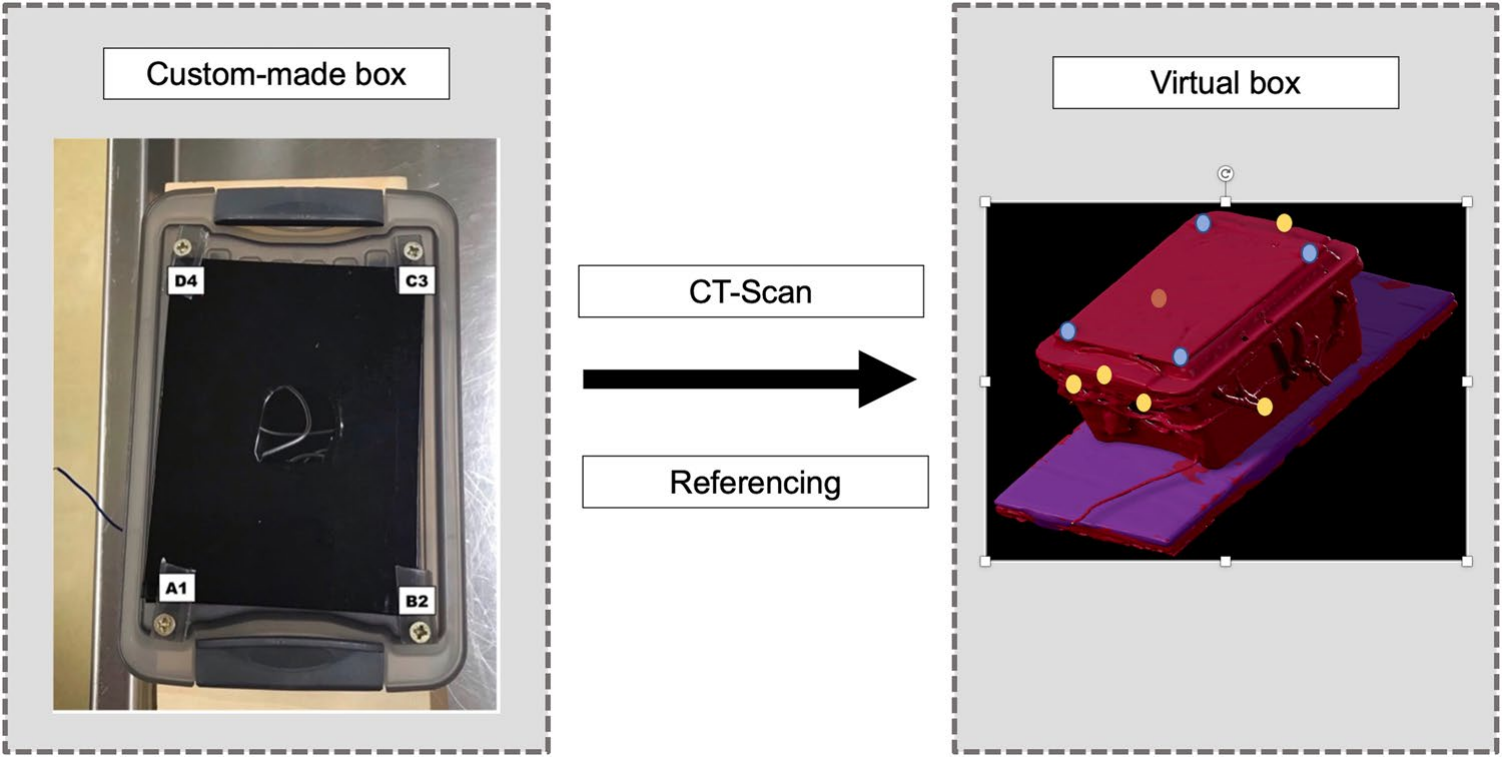
A plastic box was custom-made by fixing four metallic skews on the top of the box representing the four target points A1, B2, C3, D4. The box was scanned and referenced. Virtual objects were defined from the CT for the purpose of the neuronavigation. The blue dots represent the virtual screws (targets). The yellow dots represent the pre-defined points for the reference process of the box

For the purpose of this study, we used a 140 × 95 × 90 mm custom-made plastic box fixed to the experimental table (initially made for another experimental study [7]). Four metal cross-headed screws were fixed on the top of the box and were solidary to the box, which correspond to the 4 hallmark points (A1, B2, C3, D4) (Fig. 1).

### Neuroendoscopy

For the purpose of endoscopy, a STORZ ® endoscopy tower (Karl Storz, Tuttlingen, Germany) was used. In this study, two different rigid neuroendoscopes were used: a straight 0° and an angled 30° (length 23 cm, diameter 3.5).

### Neuronavigation, registration and neuroendoscopy

The box was first scanned (Fig. 1), and the software Elements® (Elements®, Brainlab, Germany) was used to create virtual objects such as the box borders and the cross-headed screws (targets).

The reference array, defined as the array usually fixed to the head clamp and harboring four reflective marker spheres (Brainlab, Germany), was attached to the experimental table. The box was referenced in operative condition with the NV using six pre-defined points (Fig. 1).

Because the endoscopic and navigation systems were not built to communicate, we twisted the navigation system with the use of a Bayonet Neill–Concelman connector (BNC) connector cable coupled with a BNC-HDMI connector. The video signal coming from the surgical endoscope (STORZ ®) could then be successfully integrated with the navigation Brainlab system.

**Fig. 2:**
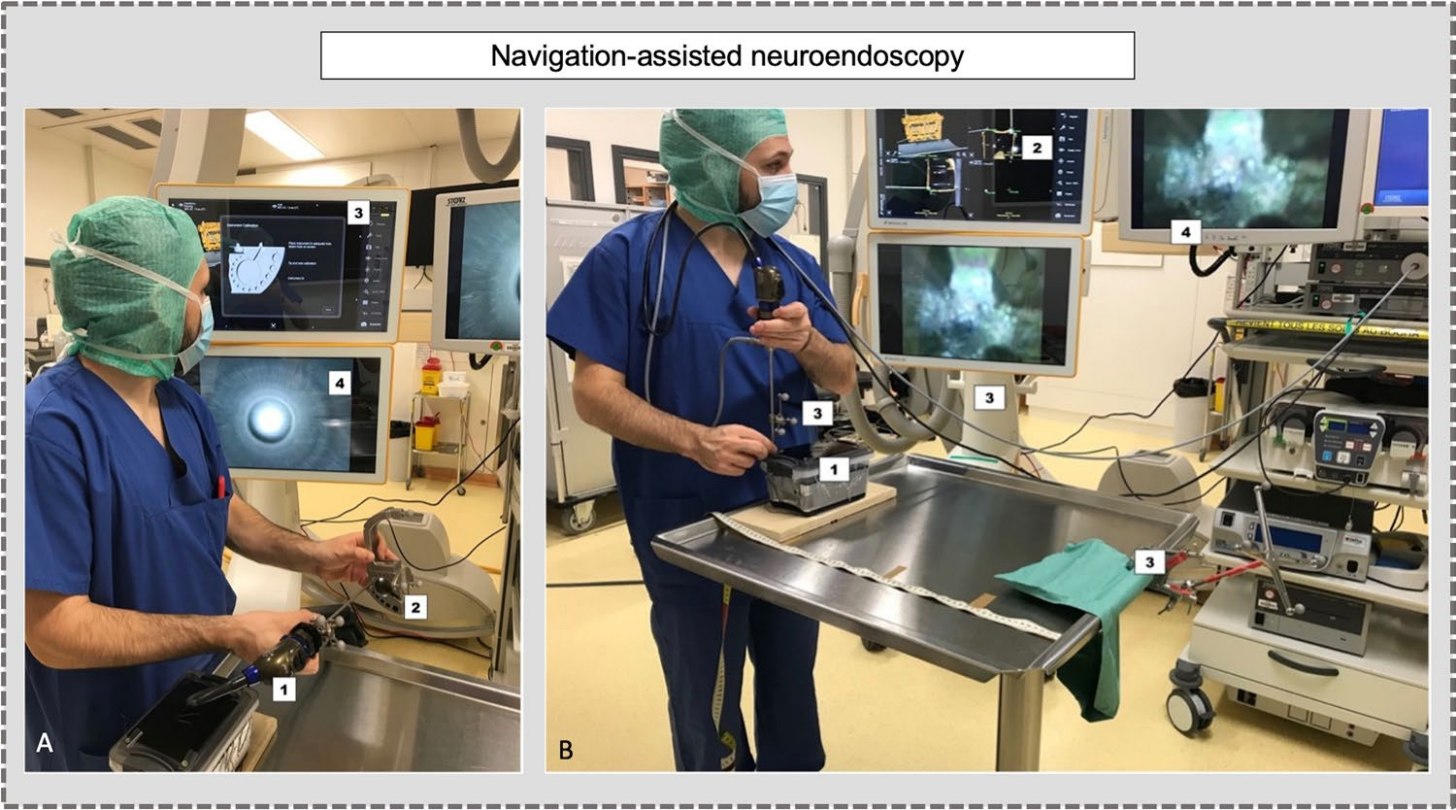
Navigation procedure of the endoscope and materials. **A**) Calibration of the endoscope [15] with the calibration instrument matrix [25]. The upper screen shows the calibration (3), and the lower screen shows the endoscope’s view. **B**) Material: [15] Box, [25] navigation view, [26] Brainlab workstation/reference array/navigation array, [10] Storz workstation

The endoscope was equipped with a navigation array, defined as the clamp with the adaptation array harboring three reflective marker spheres, and fixed on the long metallic tube of the neuroendoscope (Brainlab, Germany) (Fig. 2). To calibrate and to track the neuroendoscope, the multipurpose instrument calibration tool was used (Instrument calibration matrix®, Brainlab, Germany).

### Tasks

To assess the precision of the navigation-assisted neuroendoscopy, the center of the tip of the neuroendoscope was placed orthogonally on the center of the cross-headed screws. The position of the tip of the endoscope centered on the middle of the screw was confirmed on the midpoint of the endoscopic screen. At the same time on the NV screens, the distance in millimeters (mm) between the virtual tip of the neuroendoscope and the center of the virtual cross-headed screws (targets) was measured, defining the precision.

**Fig. 3:**
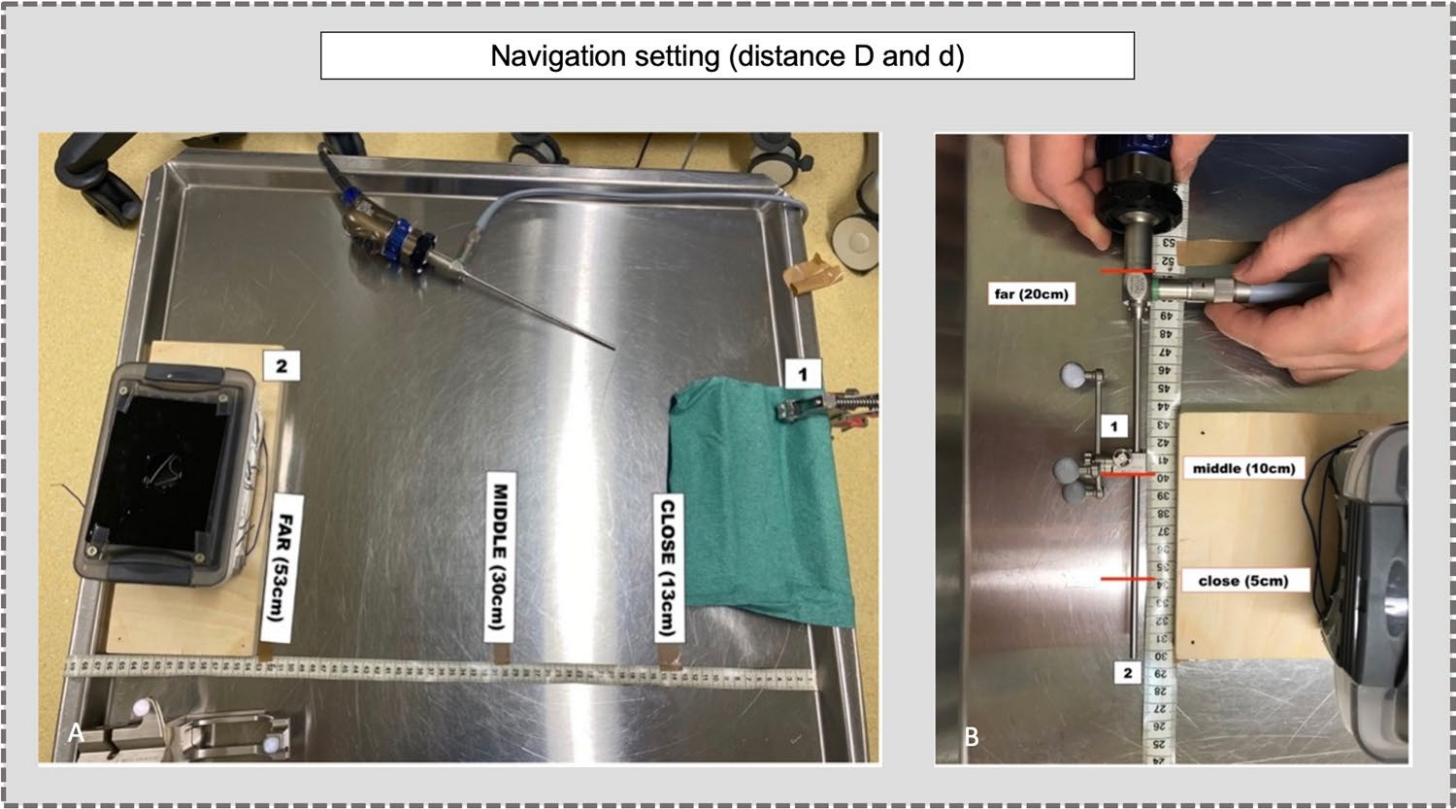
**A**) Distance *D*: positions of the reference array [15] from the box [25] (CLOSE, MIDDLE, FAR). **B**) Distance *d*: positions of the navigation arrays [15] on the neuroendoscope according to the tip [25]

The first task aimed to analyze the influence of the distances (1) between the position of the reference array and to the box (distance, D), and (2) between the position of the navigation array and the tip of the neuroendoscope (distance, *d*), and the precision (distance-to-target). For this task, a straight 0° neuroendoscope held perpendicular to the box was used. We defined arbitrary three *D*-values: CLOSE (13 cm) – MIDDLE (30 cm) – FAR (53 cm) (Fig. 3A). In the same way, we defined three *d*-values: close (5 cm) – middle (10 cm) – far (20 cm) (Fig. 3B). The nine possible combinations to measure the precision of NV were: (1) FAR/far: *D* = 53 cm and *d* = 20 cm. (2) FAR/middle: *D* = 53 cm and *d* = 10 cm. (3) FAR/close: *D* = 53 cm and *d* = 5 cm. (4) MIDDLE/far: *D* = 30 cm and *d* = 20 cm. (5) MIDDLE/middle: *D* = 30 cm and *d* = 10 cm. (6) MIDDLE/close: *D* = 30 cm and *d* = 5 m. (7) CLOSE/far: *D* = 13 cm and *d* = 20 cm. (8) CLOSE/middle: *D* = 13 cm and *d* = 10 cm. (9) CLOSE/close: *D* = 13 cm and *d* = 5 cm. For each association, the precision was measured five times for the four targets, for a total of 180 measurements. For each variation of *D*, the box was re-referenced. For each variation of *d*, the neuroendoscope was recalibrated using the instrument calibration matrix system (Brainlab, Germany) (Fig. 2).

**Fig. 4:**
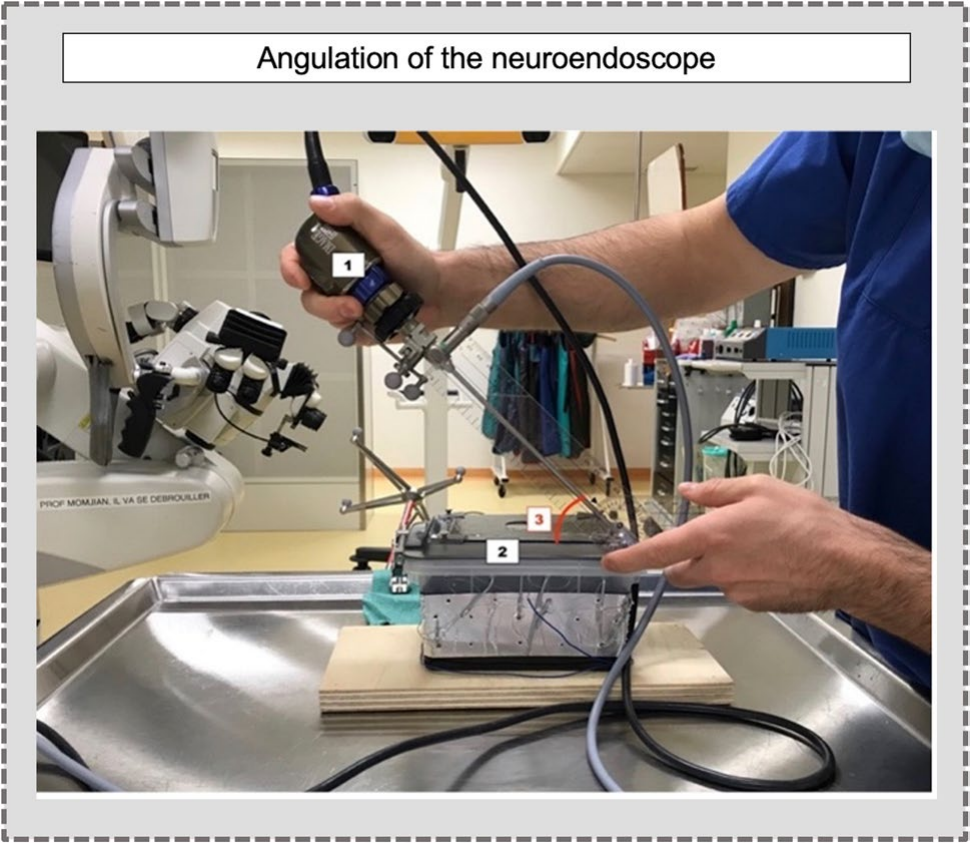
Angulation of the neuroendoscope according to the box. [15] Neuroendoscope, [25] box with the screws (targets), [26] angulation

The second task aimed to analyze the precision according to the angulation between the neuroendoscope and the box. All measures of the precision were assessed using the CLOSE/far setting. Targeting the center of the screws, the endoscope was angulated at 90° and 45° (Fig. 4). We repeated the measures either with a straight (0°) or angled endoscope (30°), defining four settings: (1) straight endoscope with 90° angle, (2) straight endoscope with a 45° angle, (3) 30° endoscope with a 90° angle, (4) 30° endoscope with a 45° angle. For each association, five trials were performed for 80 measures in total.

### Participants

All measures were performed by the first and second authors (LC and AD) reproducing surgical conditions.

### Reduction of measurement errors

Errors of precision inherent in the neuronavigation system (referencing or calibration procedures) have already been reported [7, 16, 27]. This study aims to measure the global error is term of accuracy, or the distance-to-target. This error englobes the systematic and the random errors. For this purpose, several procedures were undertaken to reduce the errors:

1. Reduction of the systematic error:
2. *Accuracy of the alignment between the endoscope optic center and the target*:
  The precision of the position of the endoscope optic center was verified with magnification on the endoscopic screen (error estimated as a twenties of millimeter). A screenshot on the navigation system was performed when the alignment was optimal, and the measures from the navigation could be assessed.
3. *Triangulation*:
  Multiple techniques were used to record the observations, for example different types of endoscope optics or different navigation setups (reference and navigation arrays).
4. *Recalibration procedure*:
  The recalibration procedure was reiterated between each measurement with a fixed position of the stereoscopic navigation camera using the multipurpose instrument calibration tool. A control of the calibration precision was performed according to the result given on the Brainlab ® navigation system. This was also inframillimetric, and the authors did not observe a tendance toward one direction of imprecision after calibration
5. Reduction of the random error:
6. *Repeated measurements*:
  For each navigation setup, five measures were performed for the four targets.
7. *Accurate sample size*:
  The sample size was calculated after analyzing the first results of precision of the opposite setups (FAR/far and CLOSE/close). Considering a type I α-error of 0.05 and a type II ß-error of 0.2, two measurements per target and per setup were needed. The authors decided arbitrary to perform five measurements per target and per setup.
8. *Controlled variables*:
  The distances *D* and *d*, the endoscope optic center or the type of endoscope was set, known and controlled for the repetitive measurements for each of the four targets.

### Statistical analyses

All statistical analyses were performed using IBM SPSS software (IBM ®, SPSS ® Statistics version 26, USA). As the variables did not follow a normal distribution (Shapiro–Wilk test *p* < 0.05), a Mann–Whitney test was used for comparisons, and, for correlations, a Spearman test was performed. We described the results in terms of median, first quartile (Q1) and third quartile (Q3). A *p* value < 0.05 was considered significant.

### Outcome measures

The primary outcome measures were (1) the median distance-to-target in millimeter (precision of navigation-assisted neuroendoscopy) according to the distance between the box and the reference array (distance *D*); (2) the median distance-to-target in millimeter (precision of navigated neuroendoscopy) according to the distance between the tip of the endoscope and the navigation array (distance *d*).

The secondary outcome was the median distance-to-target in millimeter (precision of navigated neuroendoscopy) according to the angle between the endoscope and the surface of the box and the angulation of the optics at the tip of the neuroendoscope.

## Results

### Precision of navigated neuroendoscopy

The 180 measures were all successful. Regardless the setting of *D* and *d*, the median distance between the virtual tip of the endoscope and the center of the screws using NV was 2.4 mm (Q1: 1.7; Q3: 2.9).

### Precision of navigated endoscopy and the influence of the position of the reference and navigation arrays

**Fig. 5:**
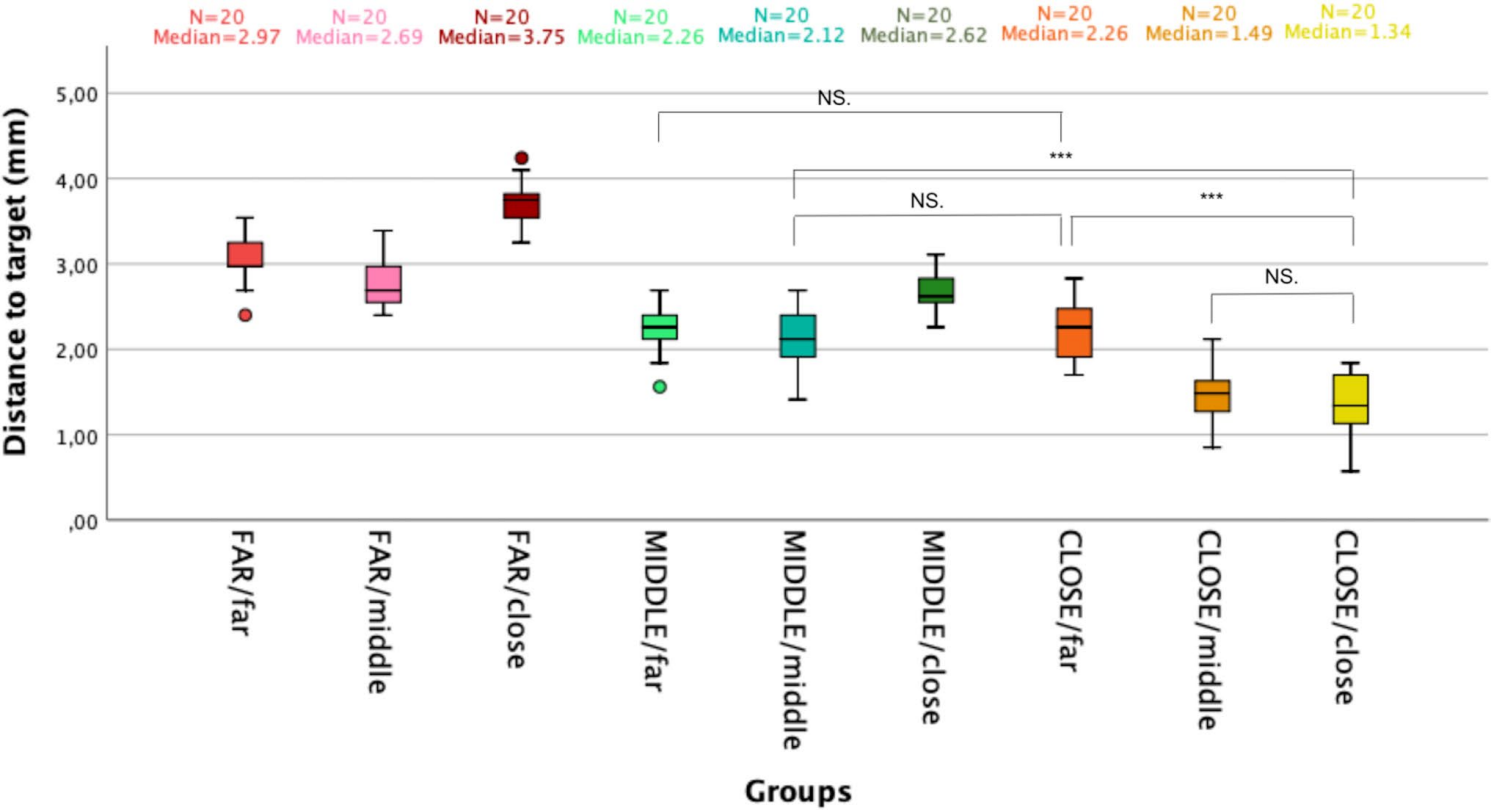
Boxplot representing the distance-to-target (precision) in millimeter, according to the setting position of the reference array to the box (FAR = 53 cm, MIDDLE = 30 cm, CLOSE = 13 cm) and the navigation array to the tip of the endoscope (far = 20 cm, middle = 10 cm, close = 5 cm)

The results are depicted as a boxplot in Fig. 5. Regarding the distance between the reference array and the box (distance *D*, FAR, MIDDLE, CLOSE), the median distance-to-target value was 3.2 mm (Q1: 2.7; Q3: 3.5), 2.4 mm (Q1: 2.1; Q3: 2.6), 1.70 mm (Q1: 1.3; Q3: 20) (*p* < 0.05), respectively.

Regarding the distance between the tip of the endoscope and the navigation array (distance *d*, far, middle, close), the median distance-to-target value was 2.4 mm (Q1: 2.2, Q3: 3.0), 2.1 mm (Q1: 1.6, Q3: 2.7), 2.6 mm (Q1: 1.7, Q3: 3.5) (*p* < 0.05), respectively.

By confronting the *D* and *d* distances, the distance-to-target for the settings FAR/far, FAR/middle, FAR/close, MIDDLE/far, MIDDLE/middle, MIDDLE/close, CLOSE/far, CLOSE/middle, CLOSE/close shows a median precision value of 3.0 mm (Q1: 3.0; Q3: 3.3), 2.7 mm (Q1: 2.6; Q3: 3.0), 3.8 mm (Q1: 3.5; Q3: 3.8), 2.3 mm (Q1: 2.1; Q3: 2.4), 2.1 mm (Q1: 1.8; Q3: 2.4), 2.6 mm (Q1: 2.6; Q3: 2.8), 2.3 mm (Q1: 1.9; Q3: 2.5), 1.5 mm (Q1: 1.2; Q3: 1.7), 1.3 mm (Q1: 1.1; Q3: 1.7), respectively (Fig. 5). No difference was found between the CLOSE/close and CLOSE/middle settings (*p* = 0.31). Precision was significantly better for the CLOSE/close setting compared to the CLOSE/far, MIDDLE/middle and MIDDLE/far setting (*p* < 0.001).

The precision was correlated with the distance of the reference array from the box (Spearman rho = 0.82): the closer the reference array to the box (distance *D*), the more precise the measures. On the contrary, no correlation of the precision of measures and the distance of navigation array from the tip of the endoscope was observed (Spearman rho = 0.040).

### Precision of navigated endoscopy and the effect of the angulation between the neuroendoscope and the target and the optic angle of the neuroendoscope

**Fig. 6:**
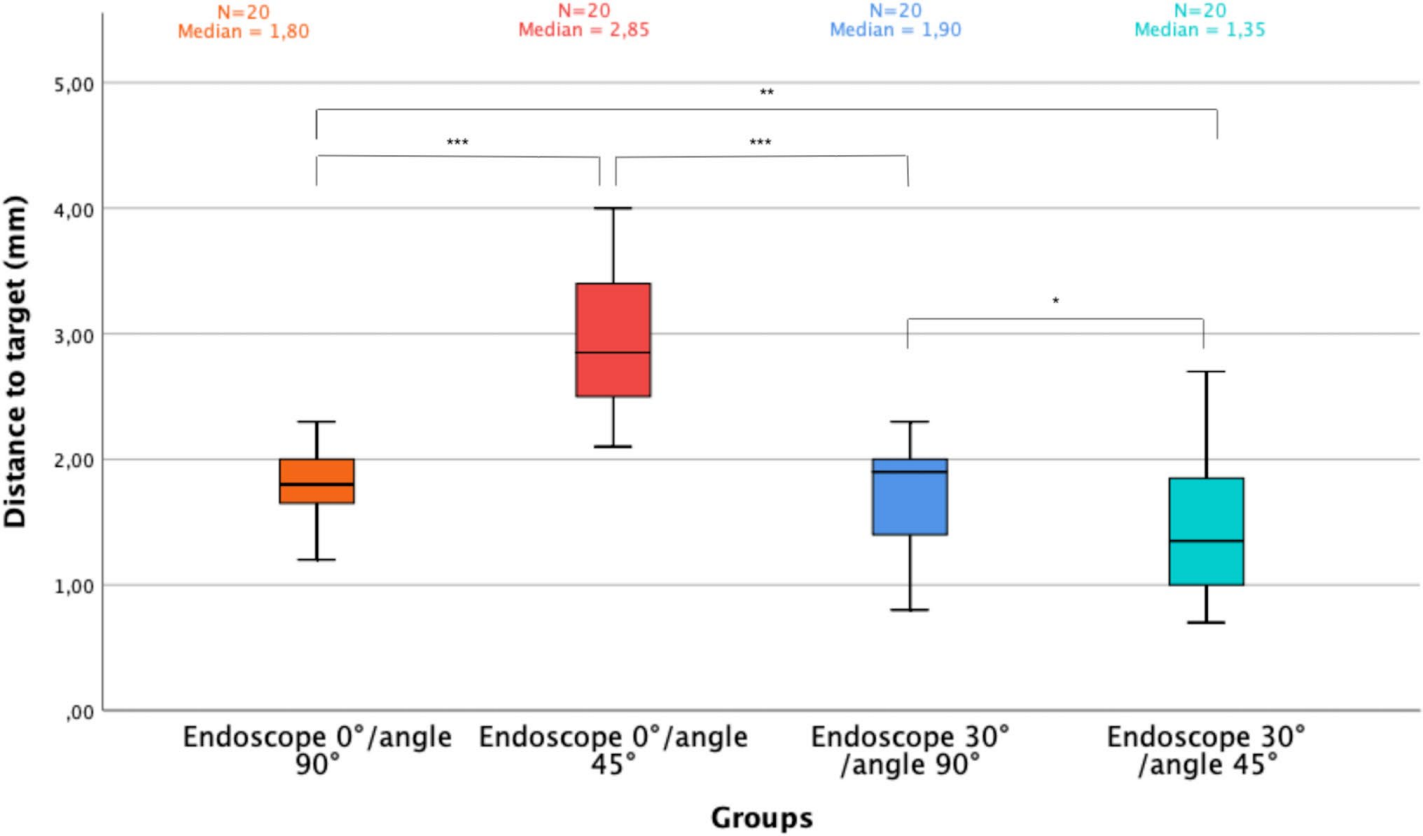
Boxplot representing precision of measures by type of endoscope and angle between box and endoscope

The results are depicted as a boxplot in Fig. 6. The 80 measurements were all successful. According to the previous results, the measures were performed according to the CLOSE/far setting. The median distance-to-target value for the 0° endoscope angled perpendicular to the target was significantly lower than with a 45° angulation (respectively: 1.8 mm (Q1: 1.7; Q3: 2.0), 2.9 mm (Q1: 2.5; Q3: 3.4)) (*p* < 0.001). Concerning the 30° neuroendoscope, the median distance-to-target value when the endoscope was perpendicular to the target was higher than at 45° angulation (1.9 mm (Q1: 1.5; Q3: 2.0) and 1.4 mm (Q1: 1.0; Q3: 1.9), respectively)) (*p* = 0.03).

No difference was found between the 0° and the 30° neuroendoscopes when the endoscopes were positioned perpendicular to the target (*p* = 0.96). In contrast, with an angulation of 45° to the target, the precision was better with a 30° neuroendoscope compared to the 0° neuroendoscope (*p* < 0.001).

## Discussion

This study is, to the best of our knowledge, the first analyzing the effect of the positioning of the reference and navigation arrays on the precision of a navigated endoscope. This has an important clinical value as the reference and navigation arrays during a navigated-endoscopic procedure are usually placed randomly or at the convenience of the surgeon. However, in the case of intracranial surgeries, the operator has to deal with millimetric anatomical structures and need an efficient and precise endoscopic navigation system [1, 4].

In this study, rigid endoscopes were referenced as navigable tools by fixing a navigation array on it and were recognized as straight rigid probes by the neuronavigation system. This setup must be differentiated from rigid probe made for a neuronavigation purpose and presenting integrated optic captors. Many studies have been already published reporting their accuracy and their efficiency [13, 22] This analysis not only confirms the possibility to navigate a rigid probe [1, 24, 29], but also presents the best setup regarding the accuracy between the reference array, the navigation array and the target.

Our analysis revealed that with respect to the distance-to-target, the most accurate setting is to place the reference array in position CLOSE (13 cm from the patient), with the navigation array in position close (5 cm from the tip of the endoscope), using a 30° endoscope with an angulation of 45° to the target. However, this theorical setting is not feasible under clinical conditions as the endoscope tip might be 7 to 10 cm deep into the brain and the navigation array would then disturb the utilization of the neuroendoscope. Consequently, this analysis showed that the second most precise setting is to set the reference array as close as possible to the patient’s head, with the navigation array placed in a far position (as distal as possible from the neuroendoscope tip) for an adult, and in a middle position for an infant, according to the skull dimension.

Lastly, we showed that the most important factor influencing the precision was the position of the reference array (short distance *D*): the shorter distance between the reference array and the box, the more accurate. This is explained by the fact that the stereoscopic camera measures angles between the reference array, the navigation array and targets. According to these angles, the navigation system estimates distances by trigonometry. Accuracy is therefore defined by the angular acuity of the system implying the ability to discriminate shorter distances when closer. The position of the navigation array influences to a lesser extent the accuracy because the angles between it and the target do not vary much (distance *d*). As well, the angulation between the endoscope and the box, and the angle of optic also influenced the precision. Endoscopic views are relatively straight, and the focus is maximal at the center of the endoscopic visual field. If the sum of the angulation of the endoscope and the angle of the optic diverges to much from 90°, a discordance according to the distance to the middle of the screw between the endoscopic focus and the virtual position of the tip of the navigated endoscope occurs.

Visualization during intracranial endoscopic procedures may be challenging, particularly if the ventricular system is filled with tumor, blood or high protein-containing fluid [9, 11, 14, 20]. Marcus et al. [20] performed a survey among endoscopic neurosurgeons in order to assess the technical challenges of neuroendoscopy. Half of the questioned surgeons reported the lack of 3D and depth perception, as well as the limited image quality. The use of NV applied to endoscopy alleviates these technical issues by providing the real-time position of the neuroendoscope and its tip on dedicated screens and according to the preoperative imagery dataset [1, 5, 8, 10, 15, 24]. Therefore, Esposito et al. [10] surveyed experienced endoscopic neurosurgeons with the aim to define the proportion of practicians performing image-guided endoscopic surgery. The authors reported that 16.6% of the intraventricular neurosurgeons use navigated neuroendoscopy in all cases and 24.4% never. In case of skull base surgery, 23.9% use NV coupled with neuroendoscopy in all cases and 18.9% never.

Two next questions arise: “for which indications should navigated neuroendoscopy be mandatory?” and “is the navigation precise enough to perform safe endoscopic surgeries?”. Rohde et al. [24] reported their experience on 121 consecutive intracranial procedures and concluded that depending on the surgical indication, navigated endoscopy offers advantages and safety: navigation offers the selection of the best trajectory for a endoscopic third ventriculostomy as it allows the surgeon to place the burr hole ideally, define an optimal trajectory and localize the perforation site in case of cyst fenestration or cystic lesion resection. The second question is answered with our work. Modern navigation systems offer a precision good enough to evolve during open intracranial procedures with safety [21, 27]. Haemmerli et al. [16] reported the inherent precision of a navigation system using a 3D-printed skull. The median distance-to-target was 3mm between the navigated target and the true target (Q1: 2mm; Q3: 4mm). Concerning endoscopic approaches, our experiment showed that the precision of navigated endoscopy was consistent with their findings during open procedure with a median distance of 2.4 mm. This distance can be optimized by adapting the position of the reference and the navigation arrays. For instance, some experimental studies concerning the precision of navigation-assisted endoscopy have been conducted for skull base indications with a distance-to-target reaching the millimetric scale in some [3, 18]. However, no detail of the navigation setup was described, nor the influence of the position of the different arrays. Furthermore, our results showed the precision varies with the type of optic and the angulation of use of the neuroendoscope. Endoscopic neurosurgeon should be aware of these variations and should be prudent while changing the working trajectory.

### Limitations

Standard neuronavigation harbors, however, one main limitation: the impossibility to recalibrate intraoperatively according to the anatomical structures. Haemmerli et al. [16] compared the precision of NV and augmented/mixed reality-guided surgery. The authors reported a better precision in terms of distance-to-target with the use of augmented reality thanks to the recalibration process. Finger et al. [11] presented in their work the first experience of augmented reality-assisted neuroendoscopy and its precision. They reported a precision of 1.2 mm, results consistent with our findings. However, no information about their navigation setup was defined.

Although we tried as possible to reproduce surgical conditions, this study is still an experimental work. Further investigations must be performed during surgeries, with the implementation of mixed reality. Furthermore, we used a standard straight rigid endoscope which did not harbor a working channel nor an instrument port. The same experiment should be also conducted with an endoscope equipped with a working channel.

### Perspectives

Because standard neuronavigation requires a cranial stabilization with the head fixed in a skull clamp, it may cause difficulties in case of surgery of young children or traumatic patients. Novel navigation technologies applied to neuroendoscopy have been developed, such as the electromagnetic navigation, without the necessity of fixing the head solidary from a reference array. Hermann et al. [17] reviewed 22 procedures using the electromagnetic navigation-assisted endoscopy on 17 patients. The authors reported an accuracy between 2.5 and 9 mm. If the precision is better using an opto-electric navigation system, the electromagnetic navigation offers promising possibilities in the future because of simplified navigation setup.

## Conclusion

The use of navigation-assisted endoscopy showed that the precision of the system is related to the position of the reference and navigation arrays, the type of endoscope and the angulation between the endoscope and the target. The reference array position is the most important factor influencing precision. In surgical conditions, the most precise and safe navigation setting is the use of a neuroendoscope with a 30° angled optic, working at a 45° angle to the target, the reference array as close as possible from the target and the navigation array at the mid distance between the tip and the connecting head on the endoscope if possible or far from the tip of the neuroendoscope.
